# Identification of Hub Genes and Potential ceRNA Networks of Diabetic Nephropathy by Weighted Gene Co-Expression Network Analysis

**DOI:** 10.3389/fgene.2021.767654

**Published:** 2021-11-01

**Authors:** Guoqing Li, Jun Zhang, Dechen Liu, Qiong Wei, Hui Wang, Yingqi Lv, Zheng Ye, Gaifang Liu, Ling Li

**Affiliations:** ^1^ Department of Endocrinology, Affiliated Zhongda Hospital, School of Medicine, Southeast University, Nanjing, China; ^2^ Department of Gastroenterology, Hebei General Hospital, Hebei, China; ^3^ Institute of Glucose and Lipid Metabolism, Southeast University, Nanjing, China

**Keywords:** diabetic nephropathy, weighted gene co-expression network analysis, hub gene, RT-qPCR, RNA regulatory pathways

## Abstract

Diabetic nephropathy (DN) is one of the most common microvascular complications in diabetic patients, and is the main cause of end-stage renal disease. The exact molecular mechanism of DN is not fully understood. The aim of this study was to identify novel biomarkers and mechanisms for DN disease progression by weighted gene co-expression network analysis (WGCNA). From the GSE142153 dataset based on the peripheral blood monouclear cells (PBMC) of DN, we identified 234 genes through WGCNA and differential expression analysis. Gene Ontology (GO) annotations mainly included inflammatory response, leukocyte cell-cell adhesion, and positive regulation of proteolysis. Kyoto Encyclopedia of Genes and Genomes (KEGG) pathways mostly included IL-17 signaling pathway, MAPK signaling pathway, and PPAR signaling pathway in DN. A total of four hub genes (IL6, CXCL8, MMP9 and ATF3) were identified by cytoscape, and the relative expression levels of hub genes were also confirmed by RT-qPCR. ROC curve analysis determined that the expression of the four genes could distinguish DN from controls (the area under the curve is all greater than 0.8), and Pearson correlation coefficient analysis suggested that the expression of the four genes was related to estimated glomerular filtration rate (eGFR) of DN. Finally, through database prediction and literature screening, we constructed lncRNA-miRNA-mRNA network. We propose that NEAT1/XIST/KCNQ1T1-let-7b-5p-IL6, NEAT1/XIST-miR-93-5p-CXCL8 and NEAT1/XIST/KCNQ1T1-miR-27a-3p/miR-16-5p-ATF3 might be potential RNA regulatory pathways to regulate the disease progression of early DN. In conclusion, we identified four hub genes, namely, IL6, CXCL8, MMP9, and ATF3, as markers for early diagnosis of DN, and provided insight into the mechanisms of disease development in DN at the transcriptome level.

## Introduction

Diabetic nephropathy (DN) is the main microvascular complication of diabetes mellitus, occurring in approximately 30–40% of patients with type 1 or type 2 diabetes, and often leads to end-stage renal disease (Alicic et al., 2017; Wang et al., 2019). Its clinical manifestations are characterized by increasing urinary albumin and serum creatine (SCR) along with decreasing estimated glomerular filtration rate (eGFR) (Zeng et al., 2019). The pathogenesis factors of DN reported in earlier studies include advanced glycosylation products, protein kinase activity, abnormal lipid metabolism and hemodynamic changes. In recent years, with the development of molecular biology, many pathways have been found to be involved in the development of DN, such as TNF signaling pathway, MAPK signaling pathway, and AGE-RAGE signaling pathway (Omote et al., 2014; Wang et al., 2019). In addition, epigenetics also plays an important role in DN development, including the post-transcriptional regulation of target genes by miRNAs and lncRNAs(Han et al., 2017). However, the underlying mechanism of DN progression is not fully understood due to the complex pathogenesis of DN.

With the remarkable evolution of bioinformatics, numerous microarray data can be used to identify hub genes, interaction networks and pathways of DN, to improve diagnosis and treatment. Weighted gene co-expression network analysis (WGCNA) is another commonly used bioinformatics analysis method in addition to differential gene expression, which can effectively explore the relationship between gene expression and clinical traits (Langfelder and Horvath, 2008). In addition, competitive endogenous RNA (ceRNA) networks may reveal new mechanisms that promote disease development in transcriptional regulatory networks (Salmena et al., 2011). Therefore, the combination of bioinformatics and epigenetics can effectively discover the hub genes and regulatory pathways related to diseases.

**FIGURE 1:**
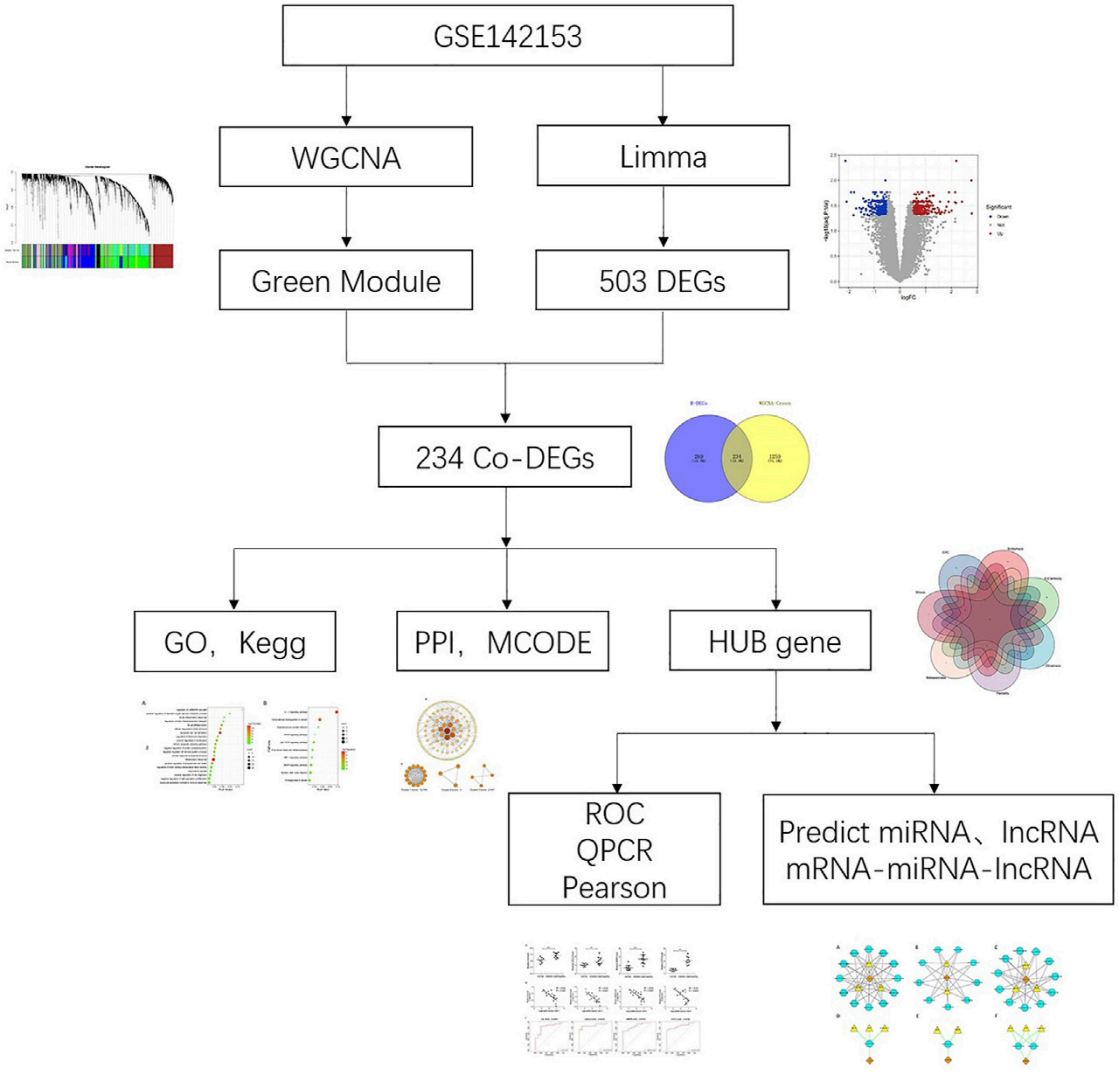
Research flow chart.

At present, most studies have focused on kidney tissue, but few have explored the effect of peripheral blood mononuclear cell (PBMC) on the pathogenesis of DN. Here, our current study analyzed the microarray datasets for PBMC in DN from the Gene Expression Omnibus (GEO) database. The differential gene expression and WGCNA were used to identify the gene-network signature and hub genes associated with DN. Subsequently, qPCR was used to verify the selected hub genes, and the correlation between their expression and eGFR was explored. Finally, based on predicted results of microRNAs (miRNAs) and long noncoding RNAs (lncRNAs), we constructed ceRNA networks to get deep understanding of its pathogenesis. We believe that this study will improve our understanding of the pathogenesis of DN and provide new insights into its treatment. The research process of this paper was showed in Figure 1.

## Methods

### Microarray Data Acquisition and Analysis

The GEO database was used to obtain microarray data from human PBMC of DN patients. Screening criteria included the following: 1) Homo sapiens Expression Profiling by array; 2) PBMC of DN patients; 3) datasets contain more than five samples. Finally, GPL6480 dataset GSE142153, which included 10 healthy control samples and 23 DN samples, was selected as test set, and the gene matrix had been normalized (supplementary Figure 1). Differentially expressed genes (DEGs) in the GSE142153 microarray were screened using the limma package in the R software (version 4.0.5) (Ritchie et al., 2015). The cut-off conditions were set to an adj.*p*.Val < 0.05, and the absolute value of log-fold change |log2FC| ≥ 0.5.

### Weighted Gene Co-Expression Network Analysis and Module Preservation

WGCNA, which constructs a scale-free network by associating gene expression levels with clinical features, is often used for a variety of systematic biological analyses. To ensure that the results of network construction were reliable, we normalized the samples first and then removed the outlier samples. The soft threshold power must be selected according to the standard scale-free networks, and the genes in the first quartile of variance were calculated by a power function. Subsequently, the adjacency matrix was transformed into a topological overlap matrix (TOM), and the corresponding dissimilarity (1-TOM) was calculated. The dynamic tree cut method was performed to identify the module by hierarchically clustering genes. A deepSplit value of 2 and a minimum size cutoff of 30 were selected as the distance measure for the resulting dendrogram. A height cutoff of 0.25 was used as the standard to merge highly similar modules. Then, we used the WGCNA package to run the module preservation function (Langfelder and Horvath, 2008).

### Finding Module of Interest

Pearson’s correlation tests were used to assess the correlation between clinical traits and modules and to identify the meaningful modules. Subsequently, we defined the correlation of the gene expression profile with module eigengenes (Mes) as a module membership (MM), and the correlation (the absolute value) between outer features and gene expression profiles were defined as the gene significance (GS). Then, we performed further analyses for the genes located in the modules of interest with the highest MM and highest GS values.

### Functional Enrichment Analysis

The genes in the module of interest were extracted and intersected with DEGs, and the results were visualized using Venn diagram. The Co-DEGs were used for further functional enrichment analysis. Gene Ontology (GO) analysis was used to identify characteristic biological attributes (Consortium, 2019). Kyoto Encyclopedia of Genes and Genomes (KEGG) pathway enrichment analysis was performed to identify functional attributes (Kanehisa et al., 2017). The significant enrichment threshold was set as *p*-value < 0.05.

### Protein-Protein Interaction (PPI) Network and Hub Genes

The PPI network was constructed based on all Co-DEGs by the online tool STRING (https://string-db.org/). Next, we downloaded the interaction information and optimized the PPI network with Cytoscape software (v3.7.2) for better visualization. Minimal Common Oncology Data Elements (MCODE) was used to identify significant gene clusters and obtain cluster scores (filter criteria: degree cut-off = 2; node score cut-off = 0.2; k-core = 2; max depth = 100). CytoHubba was used to identify significant genes in this network as hub genes (Chin et al., 2014). We used Global-based method, including seven algorithms, namely Closeness (Clo), EcCentricity (EC), Radiality (Rad), BottleNeck (BN), Stress (Str), Betweenness (BC), Edge Percolated Component (EPC), to calculate the top 20 hub genes (Ma et al., 2021). Finally, all the results were intersected to obtain the final hub genes.

### Quantitative Reverse Transcription Polymerase Chain Reaction (qRT-PCR)

Peripheral blood of healthy people and DN patients was collected from the Affiliated Zhongda Hospital of Southeast University. The eGFR level was calculated using the modified Chronic Kidney Disease Epidemiology Collaboration (CKD-EPI) equation for Asians. The patients signed the informed consent before the study, and this study has been approved by the Ethics Committee.

The PBMC was separated from peripheral blood. Subsequently, total RNA was extracted from PBMC using TRIzol, and then, its concentration and purity were assessed by nanodrop. The reverse transcription was conducted using HiScript^®^ II QRT SuperMix for qPCR (+gDNA wiper). Next, based on QuantStudio 6 Flex real-time PCR system, PCR was performed with AceQ®qPCR SYBR Green Master Mix at the temperature of 95°C for 10 min, followed by 40 cycles with the temperature of 95°C for 10 s, and 60°C for 30 s. The 2^−ΔΔCt^ method was utilized to determine the relative expression of each selected genes between DN and controls. Sequences of primers used in the study were showed in Supplementary Material.

### Prediction of Target miRNAs

We used three online miRNA databases, namely, Targetscan, miRDB, miRTarBase, to predict target miRNAs of hub genes and selected miRNAs that were found in at least two databases as the target miRNAs. The mRNA-miRNA co-expressed network based on the relationship between mRNAs and miRNAs was constructed by using Cytoscape.

### Construction of ceRNA Networks

StarBase (version 3.0) (http://starbase.sysu.edu.cn/index.php) was used to predict lncRNAs that interacted with the selected miRNAs(Li J.-H. et al., 2014). The screening criteria were: mammalian, human h19 genome, strict stringency (≥5) of CLIP-Data, and with or without degradome data. The miRNAs that were not in the Starbase were discarded, and the lncRNAs that existed in most of the predicted results of miRNAs were used as the target lncRNAs. CeRNA networks based on the interactions among mRNAs, miRNAs, and lncRNAs were constructed by using Cytoscape.

### Statistics Analysis

The R software (v4.0.5) was used to perform statistical analyses. Student’s *t*-test was used to compare the differences between the two groups. SPSS 23 was used to analyse the data and draw the ROC curve and Pearson Correlation Coefficent.

## Results

### Co-Expression Networks

**FIGURE 2:**
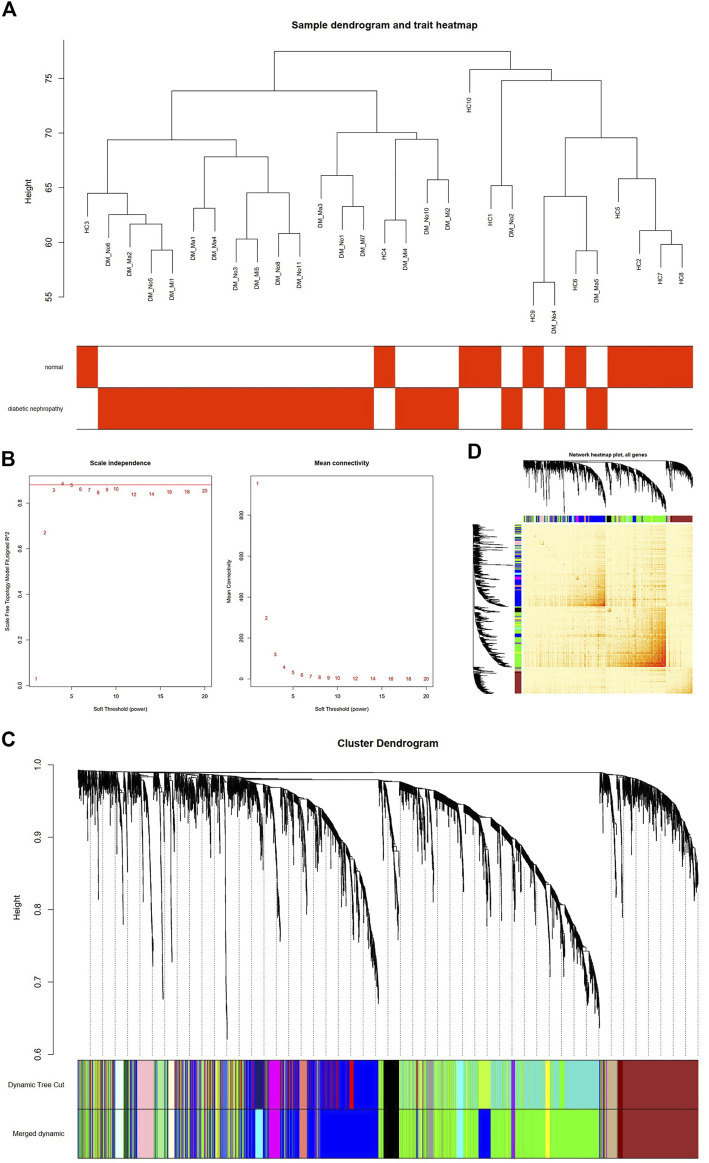
Construction of Co-Expression Network in GSE142153 by WGCNA. **(A)** Sample dendrogram and trait heatmap. **(B)** Scale independence **(Left)** and Mean connectivity **(Right)**. **(C)** The Cluster dendrogram of co-expression network modules is ordered by a hierarchical clustering of genes based on the 1-TOM matrix. Different colors represent different modules. **(D)** Network heatmap plot in the co-expression modules (The progressively saturated red colors indicated higher overlap among the functional modules.).

After the four outliers were removed, the sample cluster tree was shown in Figure 2A, and soft-threshold power of 4 was selected based on the scale-free fit index and mean connectivity values (Figure 2B). Through WGCNA analysis, 19 co-expression modules were constructed. The module with the largest number of genes was the green one, followed by the brown module, and the blue module (Figure 2C). Moreover, based on TOM, the correlation heat map between genes was shown in Figure 2D.

### Module-Trait Relationship Analysis

**FIGURE 3:**
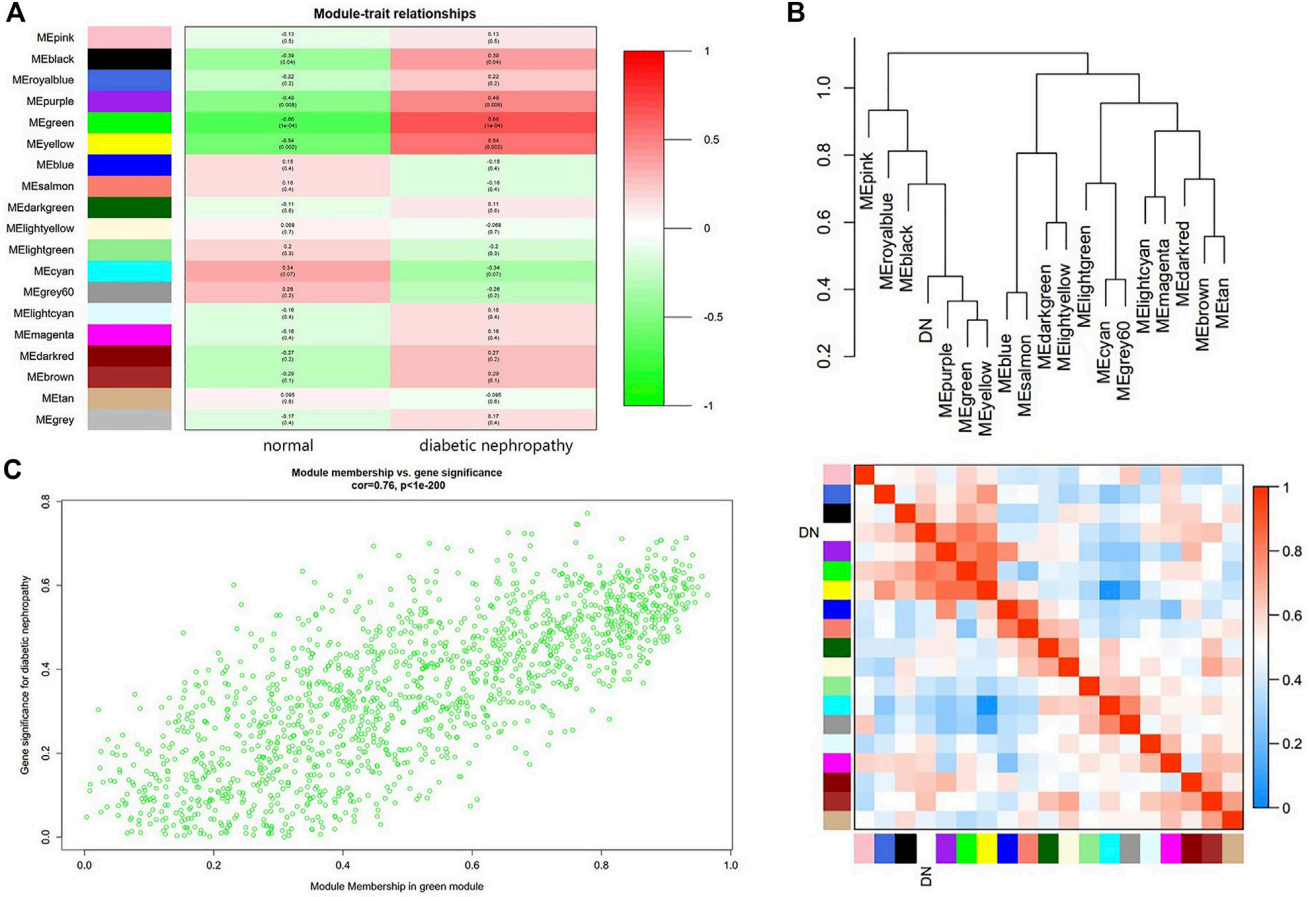
Main findings in the module-trait correlations analyses. **(A)** Module-trait relationships. Each row represents a color module and every column represents a clinical trait (normal and diabetic nephropathy). Each cell contains the correlation coefficient and corresponding *p* value. **(B)** Cluster diagram of modules **(Above)** and heatmap of trait and modules **(Below)**. **(C)** The gene significance for diabetic nephropathy in the green module (One dot represents one gene in the green module.).

Module-trait relationship analyses showed that multiple modules were related to DN, and it clearly indicated that the green module was most significantly associated with DN (Figure 3A). Moreover, DN was included in the cluster tree to uniformly make the heat map of Mes correlation. The overlap of heat maps indicated that there was some correlation between different modules (Figure 3B). In addition, Figure 3C showed the significance of these genes in the green module for DN (Figure 3C).

### Identification of the Co-DEGs of Green Module

**FIGURE 4:**
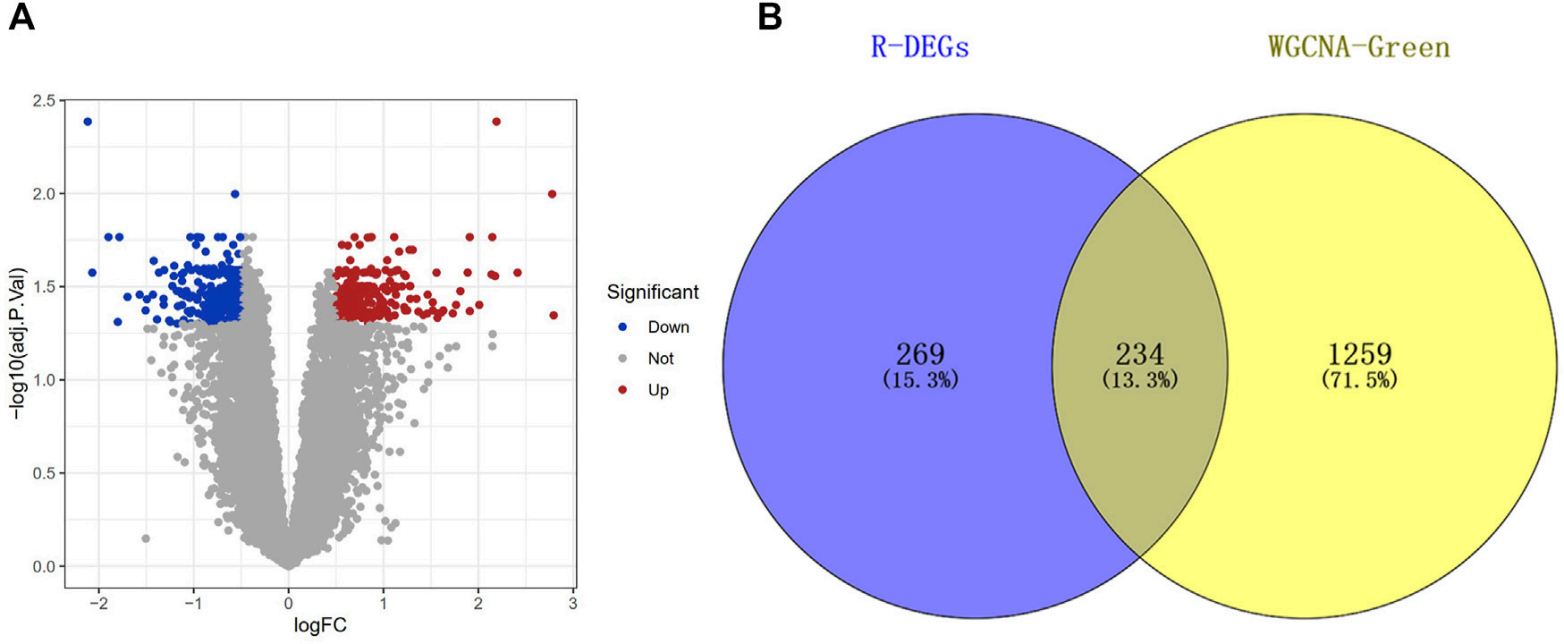
Screening of differentially expressed genes (DEGs). **(A)** Volcano of the GSE142153 dataset with the cut-off criteria of |logFC| > 0.5 and adj.*p* < 0.05. **(B)** The Venn diagram of common DEGs (Co-DEGs) in DEGs and Green module.

In order to identify the DEGs of the green module, the DEGs were identified by analyzing the dataset GSE142153. Compared with normal samples, we identified a total of 503 DEGs in the DN samples, which comprised 295 downregulated genes and 208 upregulated genes (Figure 4A). Next, The Co-DEGs were obtained from the intersection of the DEGs and the green module, and the result was showed in Figure 4B.

### Functional Enrichment Analysis of the Co-DEGs

**FIGURE 5:**
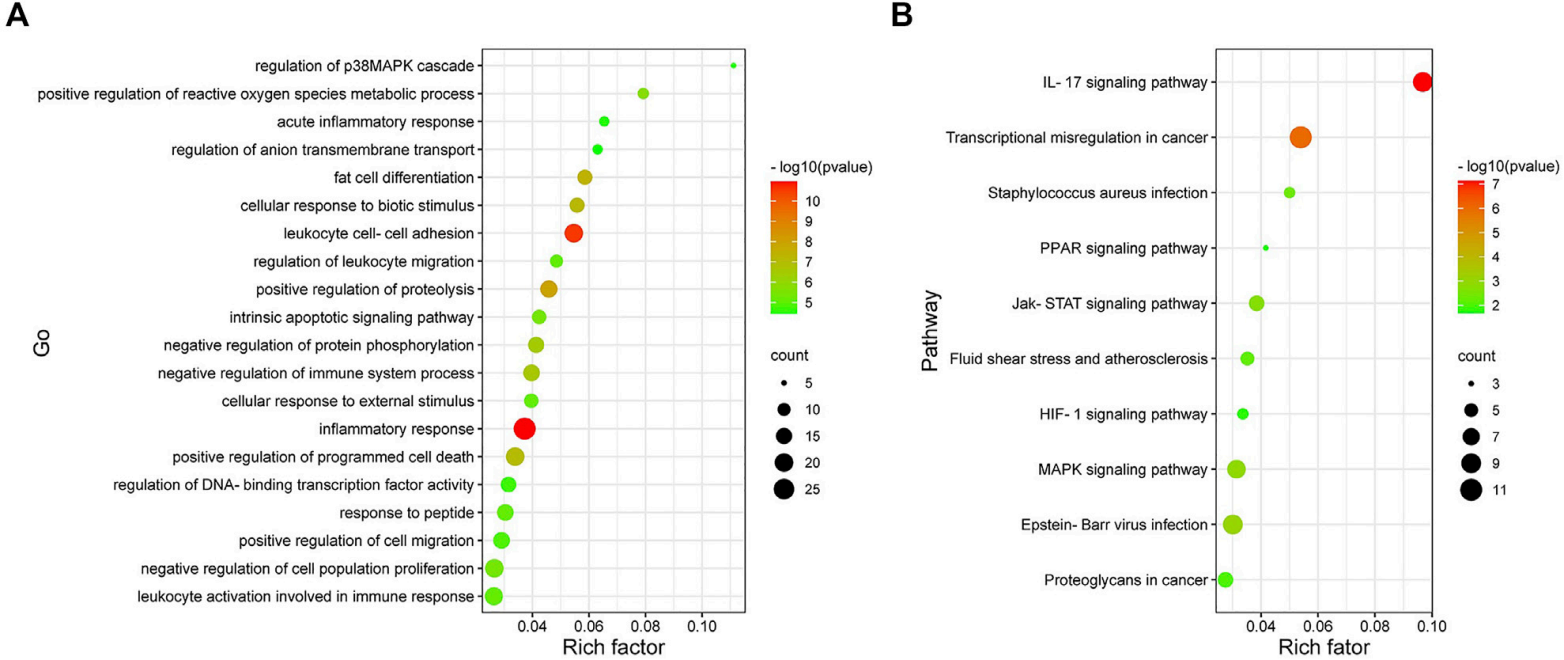
Analysis results of Co-DEGs in the Gene ontology (GO) and Kyoto encyclopedia of genesand genomes pathway (KEGG). **(A)** Results of GO enrichment analysis of Co-DEGs. The color represents the pvalue, and the size of the spots represents the gene number. **(B)** Results of KEGG pathway analysis of Co-DEGs. The color represents the *p*-value, and the size of the spots represents the gene number.

To further explore the biological function of the Co-DEGs, GO and KEGG pathway analyses were conducted (Figures 5A,B). The results of these analyses showed that, the genes were mainly enriched in biological process being involved in inflammatory response, leukocyte cell-cell adhesion, and positive regulation of proteolysis. As for the KEGG pathway, the genes were mainly enriched in IL-17 signaling pathway.

### PPI Network Construction and Gene Cluster Identification

**FIGURE 6:**
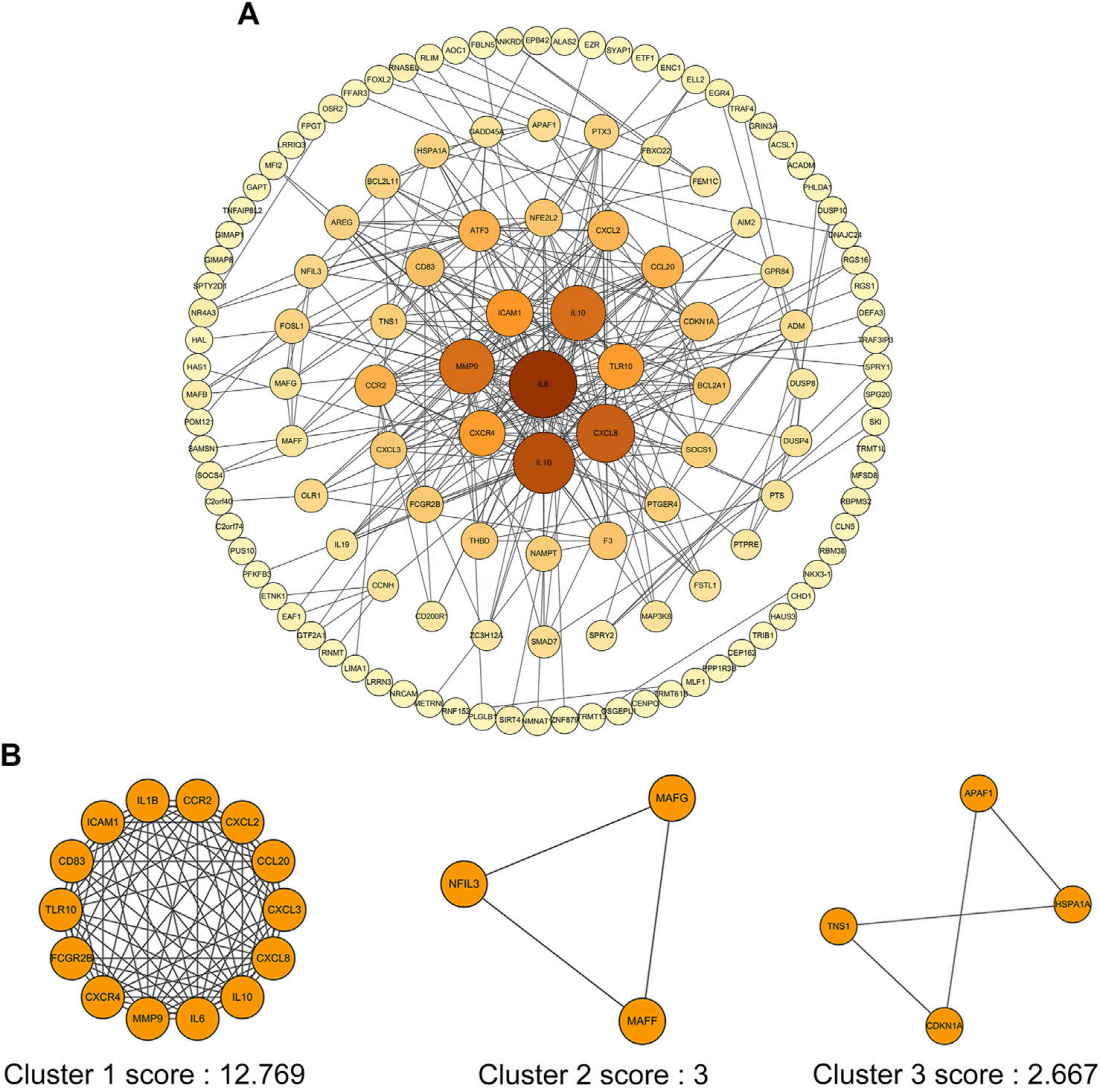
PPI network of Co-DEGs and three cluster modules extracted by MCODE. **(A)** The interaction network between proteins coded by Co-DEGs was comprised of 130 nodes and 330 edges. Each node represents a protein, while each edge represents one protein–protein association. The size of the node represents the value of Degree, and the color of the node represents Neighborhood Connectivity. **(B)** Three cluster modules extracted by MCODE.

In order to screen out the core genes from the Co-DEGs in green module, 234 Co-DEGs were uploaded to the STRING for further analysis, and 130 nodes plus 330 edges were obtained. The data file was then processed with Cytoscape as shown in Figure 6A. In addition, the size of node represented the value of Degree, and the color of node from dark to light indicated the Neighborhood Connectivity of node from high to low. MCODE was used to process the network data to identify gene clusters. There were three gene clusters, and their scores were 12.769, 3.000 and 2.667, respectively (Figure 6B).

### Hub Gene Identification and Prediction of Target miRNAs

**FIGURE 7:**
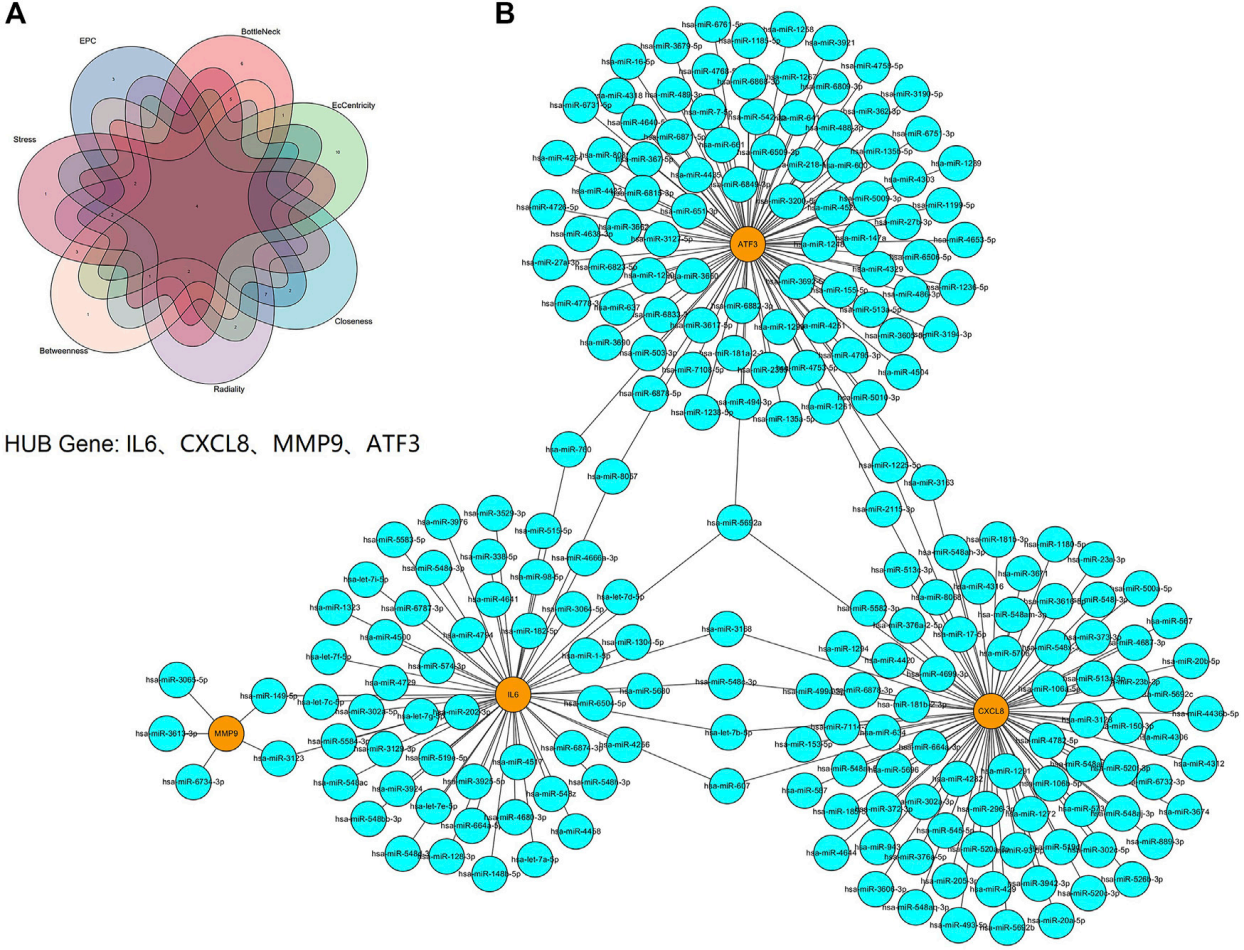
Screening of HUB genes and the co-expressed network of mRNAs and target miRNAs. **(A)** The Venn diagram of four hub genes identified by seven algorithms of cytoHubba. **(B)** The mRNA-miRNA co-expressed network was constructed by Cytoscape including 228 nodes and 237 edges. One node represents a mRNA or miRNA, while one edge represents one interaction of mRNA and miRNA. Yellow nodes represent the hub genes, and blue nodes represent miRNAs.

We used the cytoHubba plugin to identify hub genes, four hub genes were identified by intersecting the results from the seven algorithms of cytohubba including Clo, EC, Rad, BN, Str, BC and EPC (Figure 7A). These hub genes with detailed information were showed in Table 1. Next, we used three online miRNA databases to predict target miRNAs of hub genes. According to the predicted results, a co-expressed network of mRNAs and miRNAs, which comprised 228 nodes and 237 edges, was constructed by Cytoscape (Figure 7B). These hub genes were linked together by shared miRNAs.

**TABLE 1:** Four hub genes identified by seven algorithms of cytoHubba.

Gene	Description	log2FC	adj.*p*.Val	Regulation
IL6	Interleukin 6	1.003	0.039	Up
CXCL8	C-X-C motif chemokine ligand 8	1.569	0.046	Up
MMP9	Matrix metalloproteinase 9	1.195	0.032	Up
ATF3	Activating transcription factor-3	1.347	0.037	Up

FC, fold change.

### Validation and Efficacy Evaluation of Hub Genes

**FIGURE 8:**
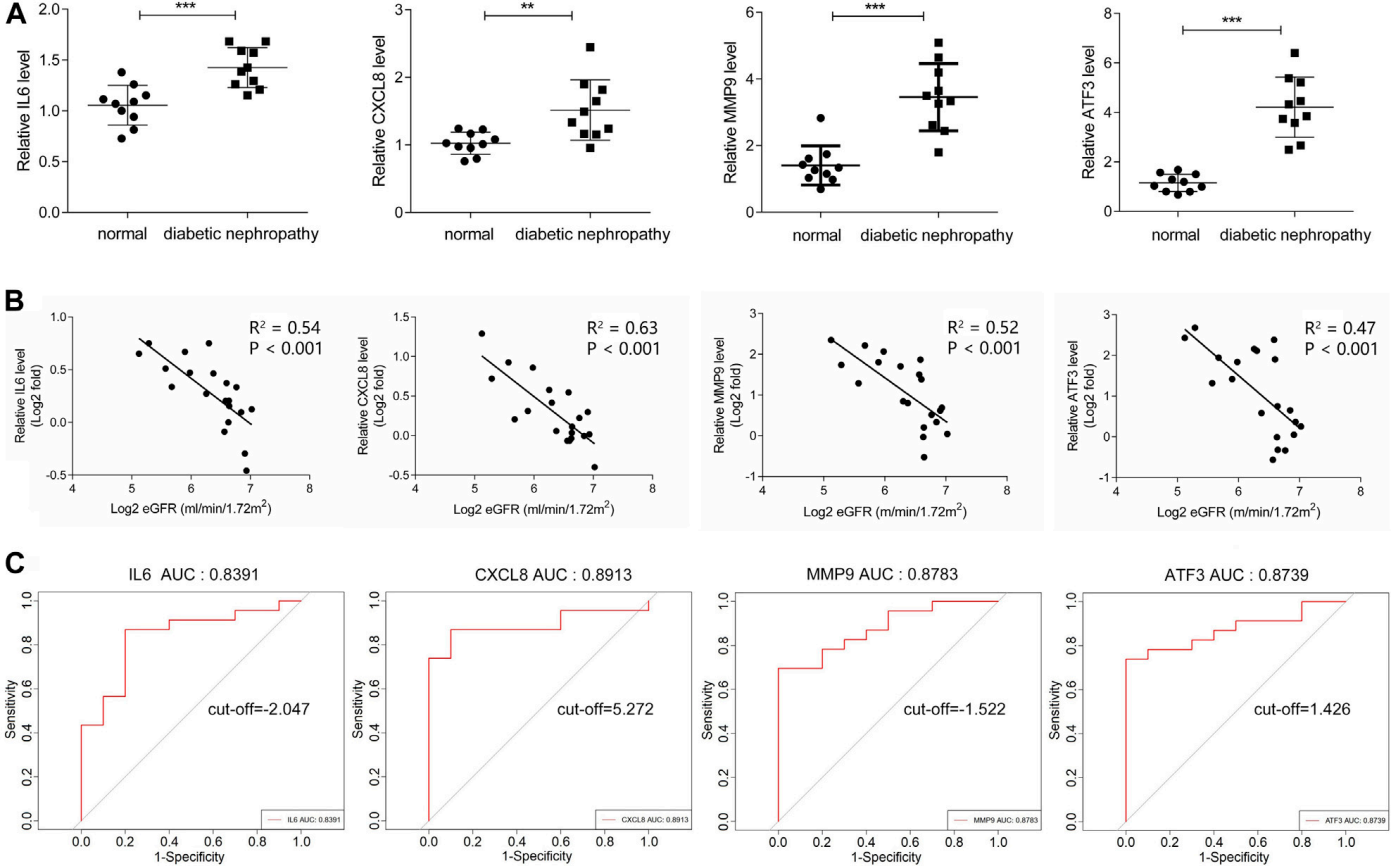
Validation and Efficacy Evaluation of HUB genes. **(A)** The expression of hub genes was detected by QPCR, compared with normal samples, all hub genes were upregulated in diabetic nephropathy samples with significance. **(B)** The expression of hub genes was negatively correlated with eGFR. **(C)** ROC curve of the hub genes including IL6, CXCL8, MMP9 and ATF3 in GSE142153.

In dataset GSE142153, the expression of four hub genes including IL6, CXCL8, MMP9 and ATF3 was significantly up-regulated in the DN patients (Table 1). What’s more, the relative expression of the above four hub genes was investigated by qPCR. As shown in Figure 8A, the relative expression of IL6, CXCL8, MMP9 and ATF3 was also significantly increased (all *p* < 0.05) in the PBMCs of DN patients compared to controls. Further exploring the association of hub genes and kidney function, the relative expression of hub genes was observed to have a negative correlation with eGFR in DN patients (Figure 8B). In addition, ROC curve was plotted and the area under the curve (AUC) was calculated to distinguish DN from controls, and every AUC of the four hub genes was greater than 0.8 in datasets GSE142153. The diagnostic value of hub genes are follows: IL6 (AUC: 0.8391), CXCL8 (AUC: 0.8913), MMP9 (AUC: 0.8783), ATF3 (AUC: 0.8739). In order to further determine the threshold to distinguish DN from control, we calculated the threshold of ROC curve of hub gene (IL6, CXCL8, MMP9 and ATF3), and their threshold values were −2.074, 5.272, −1.522, 1.426, respectively (Figure 8C).

### Prediction of Target lncRNAs and Construction of ceRNA Networks

**FIGURE 9:**
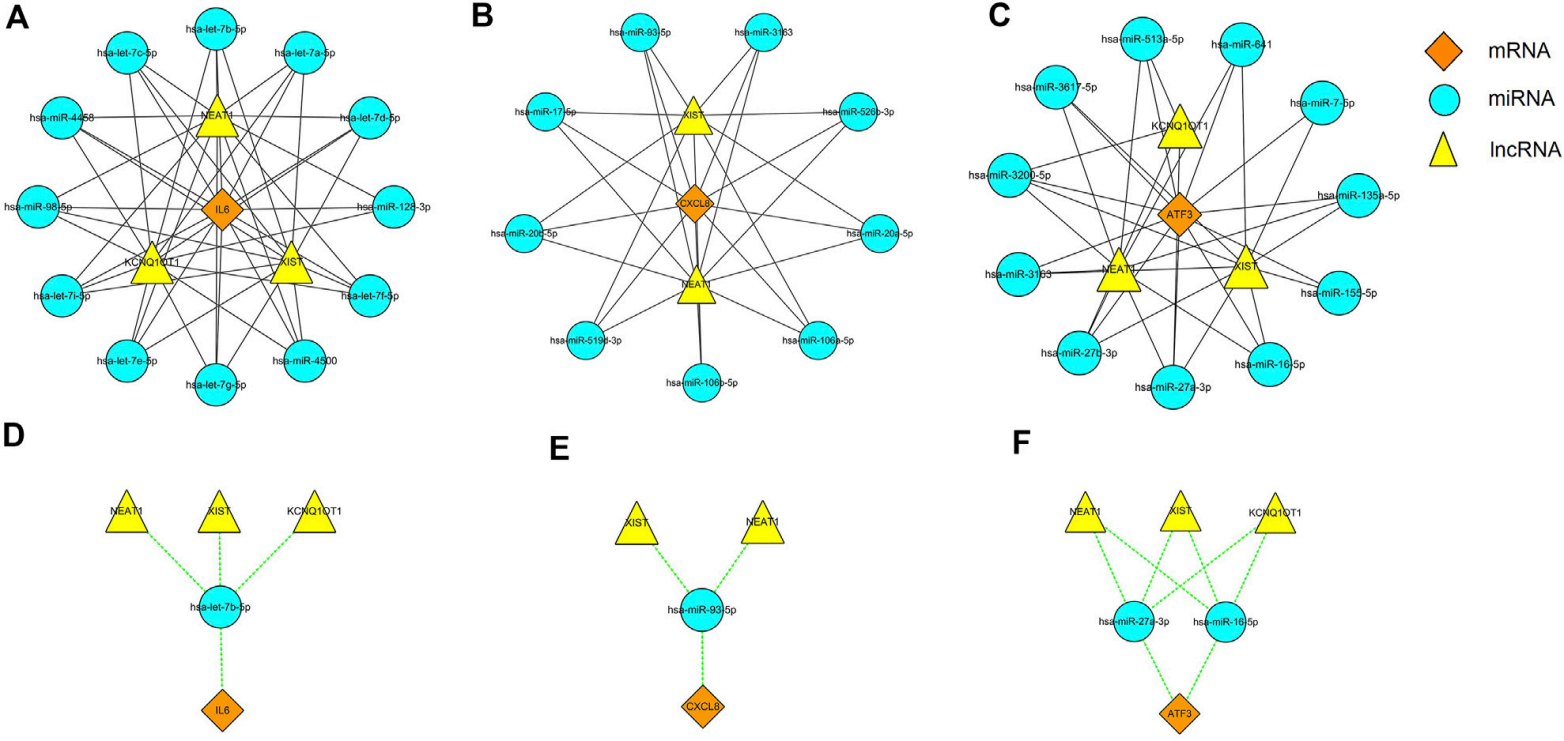
Three ceRNA networks of IL6, CXCL8 and ATF3 and the potential RNA regulatory pathways. **(A)** ceRNA network of IL6. **(B)** ceRNA network of CXCL8. **(C)** ceRNA network of ATF3. **(D)** NEAT1/XIST/KCNQ1T1-let-7b-5p-IL6. **(E)** NEAT1/XIST-miR-93-5p-CXCL8. **(F)** NEAT1/XIST/KCNQ1T1-miR-27a-3p/miR-16-5p-ATF3. Diamonds represent the hub genes, circles represent miRNAs and triangle represents lncRNAs.

It is well known that miRNAs can induce gene silencing and down-regulate gene expression by binding to mRNA. However, the upstream molecules, such as lncRNAs, can affect the function of miRNAs by binding to them, thereby upregulating gene expression. This interaction between RNAs is called a ceRNA network. we used the online database Starbase 3.0 to predict the lncRNAs that interact with the selected miRNAs. Finally, we obtained 3 target lncRNAs of the target miRNAs of IL6; 2 target lncRNAs of the target miRNAs of CXCL8; and 3 target lncRNAs of the target miRNAs of ATF3. Three ceRNA networks based on the predicted results were constructed and illustrated by Cytoscape (Figures 9A−C). Subsequently, according to the ceRNA hypothesis, we did an extensive literature search and selected four reported downregulated miRNAs and three upregulated lncRNAs in DN, for further analysis. We proposed that NEAT1/XIST/KCNQ1T1-let-7b-5p-IL6 (Figure 9D), NEAT1/XIST-miR-93-5p-CXCL8 (Figure 9E) and NEAT1/XIST/KCNQ1T1- miR-27a-3p/miR-16-5p-ATF3 (Figure 9F) might be potential RNA regulatory pathways to regulate the disease progression of early DN.

## Discussion

DN is one of the main causes of end-stage renal disease, early diagnosis and treatment can effectively improve the quality of life. Studies have long suggested that metabolic and hemodynamic changes are the underlying mechanisms of the disease. Recent studies have shown that inflammatory processes are also involved in the pathogenesis, but the exact mechanism remains unclear (Matoba et al., 2019). Our study mainly explored the effect of PBMC on the pathogenesis of DN.

In this study, a total of 503 differential genes were identified in the DN and control samples. In addition, 19 co-expression modules were obtained by WGCNA analysis. Among them, the green module was the key module mainly involved in DN, which contained a total of 1,493 genes, including 234 differential genes. In addition to the green, there were brown and blue modules involved in the DN. Thus, DN involves a complex network of gene regulation. GO and KEGG are powerful bioinformatics databases that are widely used in gene classification and signaling pathway analysis (Rue-Albrecht et al., 2016; Kanehisa et al., 2019). we applied GO analysis to elucidate the biological functions of DEGs in the green module. The results showed that the green module was mainly enriched in inflammatory response, leukocyte cell-cell adhesion and positive regulation of proteolysis. In addition, KEGG pathways were enriched in IL-17 signaling pathway, MAPK signaling pathway, JAK-STAT signaling pathway, HIF-1 signaling pathway and PPAR signaling pathway. Although DN is not an inflammatory disease, the enrichment results suggest that inflammation may be an important pathophyseological mechanism. Indeed, inflammation is increasingly recognized as central to the progression of atherosclerotic changes and microvascular complications in diabetic patients (Goldfine and Shoelson, 2017).

Through PPI network, we identified four hub genes, namely IL6, CXCL8, MMP9 and ATF3. qPCR showed that the relative expression of these hub genes in PBMC of DN was increased compared with normal groups. In addition, ROC curve showed that they were of high diagnostic value for DN. A mild decrease in eGFR has been found in some diabetic patients with normal albuminuria. Therefore, the eGFR is an important indicator for the early diagnosis of DN (Kravaritou et al., 2009). We found that these hub genes were inversely associated with eGFR, and they may be important markers of early disease and play important roles in progression. In addition, we constructed the mRNA-miRNA co-expression network and ceRNA network to clarify the pathogenesis of DN from the transcriptomic level.

IL-6 is a cytokine that is produced rapidly and briefly, primarily in response to infection and tissue damage, and thus contributes to host defense. However, continuous dyssynthesis of IL-6 has a pathological role in chronic inflammation and autoimmunity (Tanaka et al., 2014). Substantial evidence from animal and human studies supports the involvement of the IL-6 signaling pathway in the development of DN. For example, serum IL-6 levels are higher in DN patients than in the diabetic group (Taslipinar et al., 2011). Besides, serum IL-6 levels are also elevated in diabetic nephropathy mice (Liu et al., 2020). Indeed, transgenic diabetic mice with low STAT3 transcriptional activity have lower levels of IL-6 and less proteinuria in the glomeruli (Lu et al., 2009). Consistent with the present study, we found that IL6 expression was up-regulated in PBMC and JAK-STAT signaling pathway was activated. In addition, the ROC curve showed that IL6 had high diagnostic value (AUC = 0.839, cut-off = −2.047). Early studies have shown that chemokines play an important role in inflammatory kidney disease and play a key pathogenic role in the process of kidney injury (Panzer et al., 2006; Chung and Lan, 2011). CXCL8 is a typical CXC chemokine associated with recruitment and activation of neutrophils. CXCL8 is involved in the development of multiple complications, such as diabetic retinopathy, cardiovascular disease (CVD), and infection. More and more studies have shown that with the progression of DN, serum CXCL8 level gradually increases (Wong et al., 2007; Liu et al., 2018). This is consistent with our results that the expression level of CXCL8 increased with the decrease of eGFR. Furthermore, studies have shown that the elevated levels of CXCL8 in the urine of patients in early DN, while CCL2 increased in late DN, suggesting that CXCL8 may play a role in the relatively early stages of DN (Tashiro et al., 2002). Therefore, combined with the results of the ROC curve (AUC = 0.891, cut-off = 5.272), we hypothesize that CXCL8 may be a very effective biomarker for the diagnosis of early DN. MMP9 is a member of the matrix metalloproteinase family that provides homeostasis between the synthesis and degradation of the extracellular matrix to maintain the structural and functional integrity of the glomerulus (Catania et al., 2007). Studies have shown increased expression of MMP9 in the urine, serum, and renal tissues of DN, and early upregulation of MMP9 has been observed before the onset of microalbuminuria in diabetic patients. In addition, serum levels of MMP9 are inversely associated with eGFR in patients with diabetic normoproteinuria (Derosa et al., 2007; Li S.-Y. et al., 2014). Therefore, MMP9 may be an important biomarker (AUC = 0.878, cut-off = −1.522) in the early stages of diabetic nephropathy and play an important role in its progression. In our study, we also found increased expression of ATF3 in PBMC, a member of the ATF/CREB transcription factor family, which is mainly involved in endoplasmic reticulum stress response, immune response pathway and cell cycle progression. At present, there are few studies on AFT3 in DN. An animal model study showed that ATF3 overexpression aggravated podocyte injury and apoptosis *in vitro*. On the contrary, inhibition of ATF3 induction prevented podocyte injury and apoptosis *in vitro* (Zhang et al., 2018). However, ATF3 also has anti-apoptotic effects in renal tubular epithelial cells damaged by renal I/R (Li et al., 2010). Our study showed that with the decrease of eGFR, the expression of ATF3 increased. In addition, the ROC curve showed that ATF3 had high diagnostic value (AUC = 0.874, cut-off = 1.426). Therefore, we believe that ATF3 is a new and effective biomarker for the diagnosis of early diabetic nephropathy.

Furthermore, target miRNAs and the target lncRNAs of these miRNAs were predicted for IL6, CXCL8, MMP9 and ATF3, and a ceRNA network was constructed by Cytoscape. This network reveals the mechanism by which hub genes are regulated at the transcriptome level. Based on the ceRNA hypothesis, we conducted a literature search to select down-regulated miRNAs in DN for further analysis. Among the target miRNAs of IL6, CXCL8, and ATF3, the following miRNAs were down-regulated in plasma of DN: let-7b-5p, miR-93-5p, miR-27a-3p, and miR-16-5p (Pezzolesi et al., 2015; Assmann et al., 2019; Hong et al., 2021; Wang et al., 2021). In addition, lncRNA XIST, lncRNA NEAT1, and lncRNA KCNQIOT1 have been reported to be up-regulated in the serum of patients with DN (Li et al., 2020; Wang, 2020; Zhu et al., 2020). So, we speculate that NEAT1/XIST/KCNQ1T1-let-7b-5p-IL6, NEAT1/XIST-miR-93-5p-CXCL8 and NEAT1/XIST/KCNQ1T1-miR-27a-3p/miR-16-5p-ATF3 might be potential RNA regulatory pathways to regulate the disease progression of early DN. Of course, there are some limitations of our study. The sample size for analysis and validation was relatively small. In addition, due to database limitations, we did not have enough data sets to validate our results. Therefore, future studies will need to increase the sample size and conduct prospective cohort studies to further confirm our views.

## Conclusion

Our work identified four hub genes, IL6, CXCL8, MMP9, and ATF3, as potential biomarkers for the early diagnosis and treatment of DN, and provided clues to the mechanism of disease development of DN at the transcriptome level. In addition, we propose that NEAT1/XIST/KCNQ1T1-let-7b-5p-IL6, NEAT1/XIST-miR-93-5p-CXCL8 and NEAT1/XIST/KCNQ1T1-miR-27a-3p/miR-16-5p-ATF3 are potential RNA regulatory pathways that control disease progression in early DN.

## Data Availability

The datasets presented in this study can be found in online repositories. The names of the repository/repositories and accession number(s) can be found in the article/Supplementary Material.
