# Supplementary material for: Identification of Hub Genes and Potential ceRNA Networks of Diabetic Nephropathy by Weighted Gene Co-Expression Network Analysis
Source: Front Genet. 2021 Nov 1;12:767654. doi: 10.3389/fgene.2021.767654 (PMC8591079; doi:10.3389/fgene.2021.767654)
Supplement: Supplementary file 1 [file DataSheet1.zip › Additional files/supplementary materials.docx]

Table 1 The primers used in Real-time PCR (5’-3’)

| Gene | Forward Primer | Reverse Primer |
| --- | --- | --- |
| β-actin | TCCATCATGAAGTGTGACG | TACTCCTGCTTGCTGATCCAC |
| IL6 | ACTCACCTCTTCAGAACGAATTG | CCATCTTTGGAAGGTTCAGGTTG |
| CXCL8 | GACAGCAGAGCACACAAGC | GGCAAAACTGCACCTTCAC |
| MMP9 | CAGTCCACCCTTGTGCTCTTC | TGCCACCCGAGTGTAACCAT |
| ATF3 | GTGCCGAAACAAGAAGAAGG | TCTGAGCCTTCAGTTCAGCA |
