# Supplementary figures and images for: Identification of Hub Genes and Potential ceRNA Networks of Diabetic Nephropathy by Weighted Gene Co-Expression Network Analysis
Source: Front Genet. 2021 Nov 1;12:767654. doi: 10.3389/fgene.2021.767654 (PMC8591079; doi:10.3389/fgene.2021.767654)

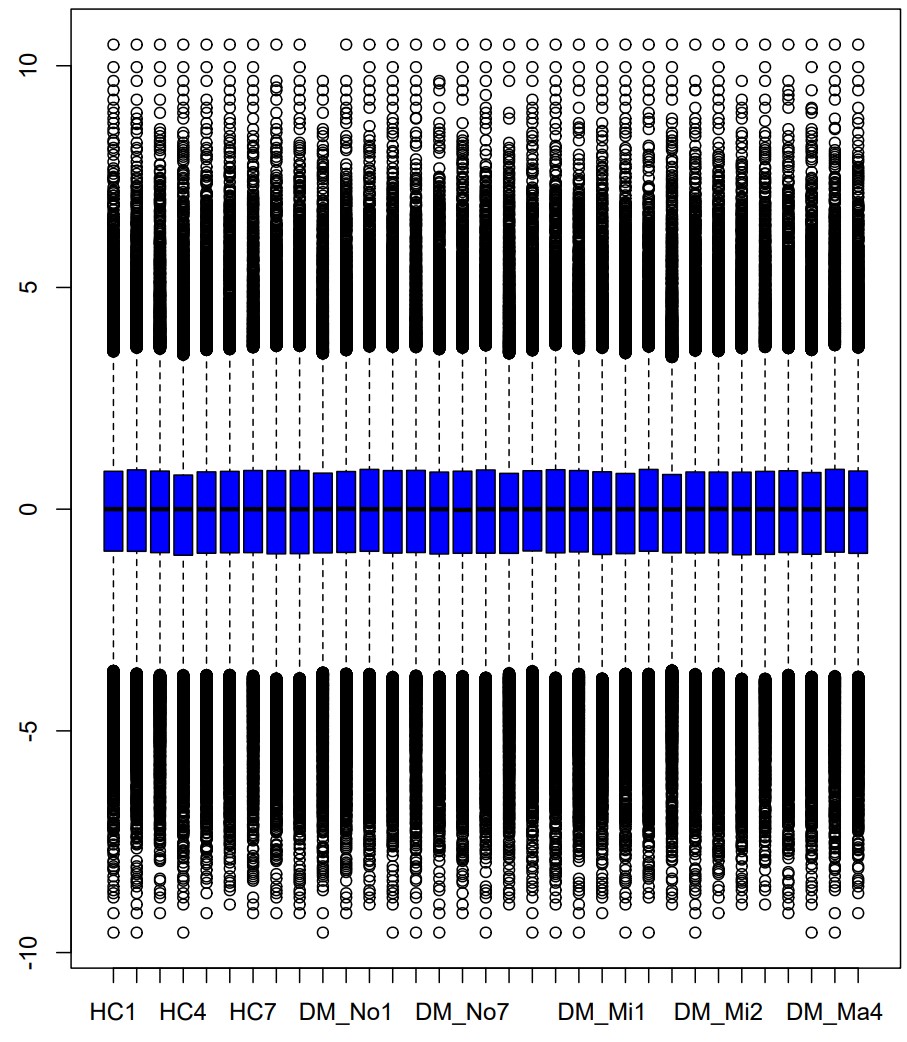

Supplement: Supplementary file 1 [file DataSheet1.zip › Additional files/supplementary Fig 1.jpg]
